# Association of the intraoperative peripheral perfusion index with postoperative morbidity and mortality in acute surgical patients: a retrospective observational multicentre cohort study

**DOI:** 10.1016/j.bja.2021.06.004

**Published:** 2021-07-03

**Authors:** Marianne Agerskov, Anna N.W. Thusholdt, Henrik Holm-Sørensen, Sebastian Wiberg, Christian S. Meyhoff, Jakob Højlund, Niels H. Secher, Nicolai B. Foss

**Affiliations:** 1 Department of Anaesthesiology and Intensive Care, Hvidovre Hospital, University of Copenhagen, Copenhagen, Denmark; 2 Department of Integrative Physiology, NEXS, University of Copenhagen, Copenhagen, Denmark; 3 Department of Anaesthesia and Intensive Care, Bispebjerg and Frederiksberg Hospital, University of Copenhagen, Copenhagen, Denmark; 4 Department of Anaesthesiology, Centre for Cancer and Organ Diseases, Rigshospitalet, University of Copenhagen, Copenhagen, Denmark

**Keywords:** cardiac output, clinical monitoring, gold-directed therapy, haemodynamics, peripheral perfusion index, postoperative complications, surgery

## Abstract

**Background:**

We hypothesised that in acute high-risk surgical patients, a lower intraoperative peripheral perfusion index (PPI) would indicate a higher risk of postoperative complications and mortality.

**Methods:**

This retrospective observational study included 1338 acute high-risk surgical patients from November 2017 until October 2018 at two University Hospitals in Denmark. Intraoperative PPI was the primary exposure variable and the primary outcome was severe postoperative complications defined as a Clavien–Dindo Class ≥III or death, within 30 days.

**Results:**

intraoperative PPI was associated with severe postoperative complications or death: odds ratio (OR) 1.12 (95% confidence interval [CI] 1.05–1.19; *P*<0.001), with an association of intraoperative mean PPI ≤0.5 and PPI ≤1.5 with the primary outcome: OR 1.79 (95% CI 1.09–2.91; *P*=0.02) and OR 1.65 (95% CI 1.20–2.27; *P*=0.002), respectively. Each 15-min increase in intraoperative time spend with low PPI was associated with the primary outcome (per 15 min with PPI ≤0.5: OR 1.11 (95% CI 1.05–1.17; *P*<0.001) and with PPI ≤1.5: OR 1.06 (95% CI 1.02–1.09; *P*=0.002)). Thirty-day mortality in patients with PPI ≤0.5 was 19% *vs* 10% for PPI >0.5, *P*=0.003. If PPI was ≤1.5, 30-day mortality was 16% *vs* 8% in patients with a PPI >1.5 (*P*<0.001). In contrast, intraoperative mean MAP ≤65 mm Hg was not significantly associated with severe postoperative complications or death (OR 1.21 [95% CI 0.92–1.58; *P*=0.2]).

**Conclusions:**

Low intraoperative PPI was associated with severe postoperative complications or death in acute high-risk surgical patients. To guide intraoperative haemodynamic management, the PPI should be further investigated.

**Editor's key points**

• Low cardiac output and poor tissue perfusion increase risk of perioperative complications
• Tissue perfusion can be measured using photoelectric plethysmographic pulse oximetry
• This study found consistent associations between a low perfusion index and postoperative complications

Postoperative complications and death are frequently associated with perioperative haemodynamic instability and perioperative hypotension has been associated with complications to major surgery. Conventional monitoring of the circulation is often based mainly on MAP and HR, but BP is also acknowledged to be an inadequate marker of organ perfusion, and as such not necessarily an effective guide for therapeutic interventions.^1^^,^^2^

With the emergence of minimally-invasive methods for cardiac output (CO) haemodynamic monitoring, goal-directed therapy (GDT) has been introduced, and may be associated with improved outcome in major elective surgery.^1^^,^^3^ However, the potential for such monitoring to improve outcome in emergency surgery needs further research.^4^^,^^5^ As a result of perioperative sympathetic or medically induced vasoconstriction, macrocirculatory parameters such as MAP and CO may be dissociated from the microcirculation and as such, these parameters may not be adequate by themselves for handling circulatory optimisation during anaesthesia.^6^ Assuming that peripheral blood flow is reduced to augment central blood volume and perfusion of vital organs during haemodynamic deterioration,^7^ a noninvasive method to detect impaired peripheral perfusion may be a relevant marker for identifying circulatory instability as demonstrated during septic and cardiogenic shock.^8^

The peripheral perfusion index (PPI), derived from the photoelectric plethysmographic pulse oximetry signal, decreases in response to hypoperfusion reflecting the ratio between the pulsatile and non-pulsatile component of the arterial waveform in the tissue.^9^ Thus, changes in peripheral perfusion, both from reduced CO and sympathetically mediated peripheral vasoconstriction to augment central blood volume, are reflected in PPI.^10^^,^^11^ Reduced peripheral perfusion has been associated with both morbidity and mortality in critically ill patients, patients with septic shock, and after acute or major elective surgery.^12^, ^13^, ^14^, ^15^ However, evaluation has only been made in relatively small cohorts of surgical patients and the association of intraoperative PPI with postoperative outcomes is inadequately described. Despite multidisciplinary efforts to improve perioperative care,^16^ patients undergoing acute major abdominal or hip fracture surgery continue to demonstrate high rates of postoperative morbidity and mortality.^17^ These patients are particularly susceptible to the effects of anaesthesia and surgery because of frailty and multiple comorbidities surgery^18^, ^19^, ^20^ and were therefore selected for this investigation of the associations between intraoperative PPI and poor outcome.

We hypothesise that haemodynamic deterioration will be reflected in a low PPI and that patients with low intraoperative PPI will have a higher risk of postoperative complications and mortality, irrespective of intraoperative MAP. Primarily we sought to evaluate the association of intraoperative PPI with severe postoperative complications or death within 30 days. Secondarily, we assessed prespecified thresholds of the intraoperative PPI in relation to outcome.^21^

## Methods

### Design, setting, and participants

This retrospective observational cohort study included all consecutive patients who had acute hip fracture or major abdominal surgery from November 1, 2017 to October 31, 2018 at Hvidovre and Bispebjerg University Hospitals, Copenhagen, Denmark. The study was registered at Clinical Trials October 15, 2018 (NCT03757442) and the protocol and statistical analysis plan was peer-reviewed and published; perioperative data were collected after defining exposure and outcome variables.^21^ Reporting is according to the Strengthening the Reporting of Observational Studies in Epidemiology (STROBE) statement.

We included patients ≥18 yr old undergoing neuraxial or general anaesthesia for either acute hip fracture surgery (arthroplasty, intramedullary nailing or screws) or acute abdominal surgery (laparoscopy or explorative laparotomy as a result of ileus, any perforation of viscera, or any ischaemic condition of the gut). Patients were identified from the hospital's electronic medical records via specific procedural codes via the civil registration number, which is a unique identifier assigned to all Danish citizens at birth. Patients were excluded if surgery was not performed because of the abovementioned pathology, if there was no registration of the intraoperative PPI in the anaesthesia chart, if the civil registration number was foreign, if patients were lost to follow-up, or if the patient was previously included in the cohort.

The regional research ethics committee approved the study (H-18058705) and the board of directors at the two involved hospitals and departments approved access to hospital medical records. Management and storage of data were approved by the Regional Data Protection Agency (WZ 18049692 and WZ 17038300-1018-77).

Entry into the electronic patient records was logged by special identification numbers assigned to the working group obtaining data and according to law, all data are anonymous. All data were collected from the electronic medical records. No patient was exposed to any inconvenience in relation to the study and the study did not impact treatment of the involved patients, which was why we did not involve patients or the public in designing the study or collection, management, analysis, or interpretation of the data.

### Exposures

Intraoperative MAP, HR, SpO_2_, temperature (Temp), and PPI were obtained continuously from the patient monitor (Philips IntelliVue MP50, Koninklijke Philips, Eindhoven, the Netherlands) as part of clinical routine. PPI was obtained using the photoelectric plethysmographic pulse oximetry signal from the index finger. BP was either measured with the oscillometric noninvasive technique or via radial arterial cannulation and data from the latter was used when available. We obtained PPI, MAP, HR, SpO_2_, and Temp as 15 min averages intraoperatively, defined as the period from induction of anaesthesia to the last suture.

We defined pragmatic thresholds for low values of PPI to be 0.5 and 1.5 and MAP 65 mm Hg for assessment of the association of combinations of low/normal MAP and low values of PPI with outcome.^10^^,^^22^ We considered mean intraoperative PPI to be the primary exposure variable.

### Other exposures

Patient characteristics and intraoperative variables such as type of surgery, anaesthetic method, use of inotropes and vasoactive medication, and i.v. administered fluids, and comorbidities were obtained from the electronic patient record. Comorbidity was ranked according to (1) ASA, (2) WHO/ECOG/Zubrod score that assesses the patient's ability to carry out daily activity: 0 (unrestricted) to 4 (bedridden), and (3) the Charlson Comorbidity Index that categorises comorbidity based on International Classification of Diseases diagnosis codes.

### Outcome measures

The Clavien–Dindo Classification was used to register surgical complications; severe complications were defined as Class III–V, which is those requiring surgical, endoscopic, or radiological intervention, and life-threatening complications requiring ICU admission, or death. We defined the primary outcome to be severe postoperative complications or death (Clavien–Dindo Class ≥III) within 30 days. All-cause mortality at postoperative Day 30 and Day 90 was obtained by review of the patient records and via the civil registration number, ensuring 100% follow-up.

### Data collection

Data were obtained by review of patient records and from the anaesthesia charts, and all data was recorded and managed using Research Electronic Data Capture (REDCap) hosted by The Capital Region of Denmark. Data entry was done by MA and ANWT from February 1, 2019 to February 2020.

### Statistical analysis

According to the prespecified statistical analysis plan^21^ the sample size calculation was based on previous studies^23^^,^^24^ suggesting that the overall probability of severe postoperative complications or death within 30 days would be 40%. We expected to include approximately 2300 patients in the predefined inclusion period, which, using Whittemore's formula, would allow for detection of an OR of 1.1 using a two-tailed test with a significance level of 5% and a power of 80%.

Descriptive statistics were applied; data are presented as median with inter-quartile range [IQR], mean (standard deviation, sd), or frequencies with percentages (%) where appropriate. Differences in baseline characteristics were stratified by anaesthetic method (general anaesthesia *vs* neuraxial anaesthesia) and analysed using the χ^2^ test, the independent sample *t*-test, or the Wilcoxon rank sum test where appropriate.

To evaluate the association of intraoperative PPI and low values of PPI (PPI ≤1.5 and PPI ≤0.5) with the primary outcome we used logistic regression. Univariable logistic regression was performed to evaluate possible confounding between outcome and risk factors. Risk factors with significant physiological and statistical association with outcome were assessed one by one in a multivariable model with PPI as the primary exposure variable and Clavien–Dindo Class ≥III as the dependent variable, evaluating each risk factor for potential confounding by assessing the estimate.

In the final multivariable model (PPI as primary exposure and Clavien–Dindo Class ≥III as the dependent variable), we included risk factors with significant univariable association to outcome and effect on the estimate: age, sex, type of surgery (abdominal *vs* hip fracture), type of anaesthesia (general anaesthesia *vs* neuraxial anaesthesia), intraoperative infusion of (any) vasoactive medication, administered fluids (crystalloids and colloids per 100 ml), blood loss (per 100 ml), and comorbidities using ASA physical status, Zubrod Score and, Charlson Comorbidity Index.

To evaluate the associations in subgroups of patients with mean intraoperative PPI ≤0.5 and 1.5, we used the same multivariable model with PPI ≤0.5 and PPI ≤1.5 as the primary exposure variables. To evaluate the association of PPI and 30-day mortality, we used the same multivariable model with 30-day mortality as the dependent variable.

To evaluate the association in subgroups of patients with MAP ≥65 mm Hg (normotensive) and MAP <65 mm Hg (hypotensive), we carried out logistic regression for the association of intraoperative PPI with the primary outcome adjusting for interaction between MAP and PPI. For each model, calibration plots were drawn, and to evaluate the goodness-of-fit and the observed *vs* expected outcome for each model, we applied the Hosmer–Lemeshow test.

*Post hoc*, we evaluated the estimated probability (%) of severe postoperative complications or death within 30 days according to cumulated intraoperative time below the specified PPI thresholds from the logistic regression model. We assessed the predictive accuracy, sensitivity, and specificity of very low PPI (≤0.5) for Clavien–Dindo Class ≥III and 30-day mortality.

To assess the best PPI cut-off in the data, we applied receiver operating characteristic (ROC) curve analysis maximising sensitivity and specificity using Youden's index.

The study protocol planned to handle missing data on exposure and outcome variables exceeding 10%, however, data entry was complete for the cohort with no variables exceeding 10% missing data. A two-sided *P*-value <0.05 was considered statistically significant and all analysis were conducted by use of statistical software from RStudio (2016), Integrated Development for R (RStudio, Inc., Boston, MA, USA).

## Results

### Baseline characteristics

**Fig 1:**
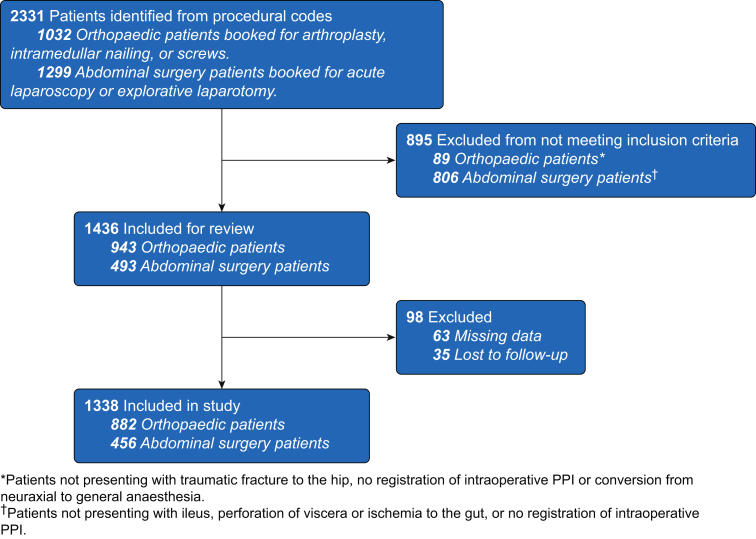
Patients undergoing acute major abdominal or hip fracture surgery from November 2017 to October 2018 at Hvidovre and Bispebjerg University Hospitals, Copenhagen, Denmark. PPI, peripheral perfusion index.

Between November 1, 2017 and October 31, 2018, we included 1338 acute surgical patients; 456 (34%) having acute high-risk abdominal surgery (AHA), and 882 (66%) presenting with hip fracture (Fig. 1). Seven hundred and one (58%) patients underwent general anaesthesia and 567 (42%) underwent neuraxial anaesthesia. Patients were 76 [66–85] (median [IQR]) yr old and 526 (39%) were male. Preoperative PPI, MAP, and HR were, mean (sd), 2.6 (2.6), 87 (18) mm Hg, and 85 (17) beats min^−1^, respectively. Overall, 755 (56%) presented with ASA Class ≥3.

Intraoperative PPI was 3.9 (2.8) in patients undergoing general anaesthesia and 2.5 (2.1) in patients undergoing neuraxial anaesthesia (*P*<0.001). One hundred and three (8%) patients had PPI ≤0.5 and 397 (30%) a PPI ≤1.5. MAP was 69 (9) mm Hg for patients undergoing general anaesthesia and 68 (9) mm Hg for patients undergoing neuraxial anaesthesia (*P*<0.001), whereas overall 448 patients (34%) had a mean intraoperative MAP ≤65 mm Hg.

A total of 325 patients (24%) experienced severe postoperative complications or death within 30 days (Clavien–Dindo Class ≥III) with 241 (74%) of them undergoing general anaesthesia and 84 (26%) neuraxial anaesthesia (*P*<0.001). Thirty-day overall mortality was 139 (10%). Intraoperative haemodynamic variables, anaesthetic methods, vasoactive medication, fluid use, and outcome are shown in Table 1.

**Table 1:** Intraoperative haemodynamic variables, anaesthetic methods, vasoactive medication, fluid use, and outcome in acute high-risk abdominal and hip fracture patients.

	All *n*=1338	General anaesthesia *n*=771	Neuraxial anaesthesia *n*=567	*P*
Peripheral perfusion index	3.3 (2.6)	3.9 (2.8)	2.5 (2.1)	<0.001
Mean PPI ≤0.5	103 (8)	34 (4)	69 (12)	<0.001
Mean PPI ≤1.5	397 (30)	165 (21)	232 (41)	<0.001
Cumulated min with PPI ≤0.5	20 (38)	13 (33)	28 (43)	<0.001
Cumulated min with PPI ≤1.5	54 (61)	42 (59)	71 (60)	<0.001
MAP (mm Hg)	69 (9)	68 (9)	71 (10)	<0.001
Mean MAP ≤65	448 (34)	271 (35)	177 (31)	0.2
Cumulated min with MAP ≤65	62 (52)	67 (52)	56 (51)	<0.001
HR (beats min^−1^)	77 (14)	76 (15)	79 (13)	<0.001
SpO_2_ (%)	99 [97–100]	99 [98–100]	98 [96–100]	<0.001
Temp (°C)	36.7 (0.7)	36.7 (0.7)	36.8 (0.7)	0.2
Haemoglobin (mmol L^−1^)	6.9 (1.3)	7.0 (1.3)	6.6 (1)	<0.001
Lactate (mmol L^−1^)	0.9 [0.7–1.3]	0.9 [0.7–1.4]	0.9 [0.6–1.2]	0.01
Duration of surgery (min)	82 [60–118]	92 [64–135]	76 [58–96]	<0.001
Volatile anaesthetic	208 (27)	208 (27)	–	–
Total intravenous anaesthesia	561 (73)	561 (73)	–	–
Thoracic epidural blockade	284 (37)	284 (37)	–	–
Sedation	389 (69)	–	389 (69)	–
Inotropes	47 (2)	45 (6)	2 (0.4)	<0.001
Infusion vasopressor∗	502 (38)	392 (51)	110 (19)	<0.001
Blood products	213 (16)	129 (17)	84 (15)	0.4
Fluids^†^ (ml)	1301 (900)	1350 (1012)	800 (514)	<0.001
Blood loss (ml)	100 [0–300]	75 [0–250]	200 [50–300]	<0.001
Clavien–Dindo Class ≥III	325 (24)	241 (31)	84 (15)	<0.001
30-Day mortality	139 (10)	97 (17)	42 (7)	<0.001
90-Day mortality	234 (18)	148 (19)	86 (15)	0.07
LOS (days)	8 [5–12]	8 [5–13]	8 [5–11]	0.06
Readmissions	387 (30)	228 (31)	159 (29)	0.4

Data are presented as [inter-quartile range], mean (standard deviation) or *n* (%). ∗Phenylephrine, noradrenaline, or both. ^†^Ringer's acetate, Ringer's lactate, NaCl, HA, human albumin; Voluven®, or any combination of these. LOS, length of stay; SpO_2_: peripheral capillary oxygen saturation; Temp, temperature.

**Fig 2:**
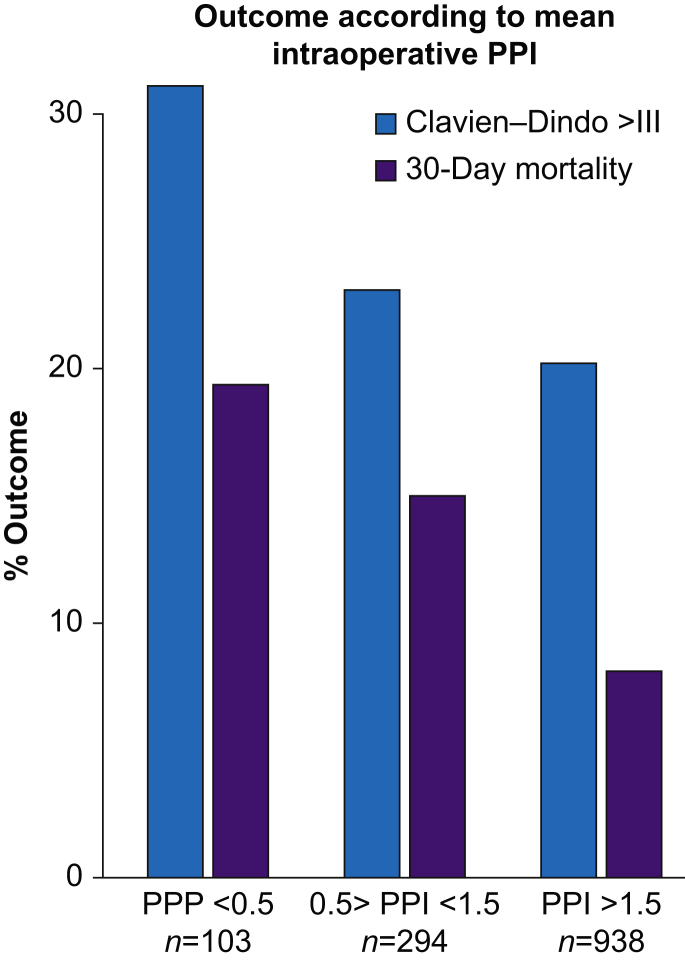
Percentage Clavien–Dindo Class ≥III and 30-day mortality according to mean intraoperative PPI in patients undergoing acute high-risk abdominal or hip fracture surgery. PPI, peripheral perfusion index.

Patients with PPI ≤0.5 had a 19% mortality within 30 days in contrast to patients with an intraoperative PPI>0.5 in whom 30-day mortality was 10%, *P*=0.003. The 30-day mortality was 16% *vs* 8% in patients with a PPI ≤1.5 and >1.5, respectively (*P*<0.001). Outcomes according to mean intraoperative PPI are shown in Fig 2.

### Association of PPI with outcome

In evaluating association of PPI with the primary outcome of severe postoperative complications or death within 30 days, the crude OR for the cohort was 1.07 (95% CI 1.02–1.13; *P*=0.01). The univariable association of PPI ≤0.5 and PPI ≤1.5 with outcome was 1.67 (95% CI 1.08–2.55; *P*=0.02) and 1.36 (95% CI 1.04–1.78; *P*=0.002), respectively, for the whole cohort. MAP ≤65 mm Hg did not present a significant association with the primary outcome in the univariable model (OR 1.21; 95% CI 0.92–1.58; *P*=0.2). The univariable association of other potential risk factors, stratified by anaesthetic method with the primary outcome, are shown in Table 2.

**Table 2:** Univariable association between potential risk factors/confounders with the primary outcome of severe postoperative complications: Clavien–Dindo Class ≥III within 30 days in acute high-risk abdominal and hip fracture patients.

	All *n*=1338	*P*	General anaesthesia *n*=771	*P*	Neuraxial anaesthesia *n*=567	*P*
PPI, per 1.0 decrease in mean PPI	1.07 (1.02–1.13)	0.01	1.13 (1.07–1.21)	<0.001	1.13 (1–1.29]	0.07
Mean PPI ≤0.5, yes	1.67 (1.08–2.55)	0.02	3.33 (1.66–6.84)	0.001	1.72 (0.89–3.15)	0.09
Mean PPI ≤1.5, yes	1.36 (1.04–1.78)	0.002	2 (1.40–2.85)	<0.001	1.45 (0.91–2.31)	0.1
PPI ≤0.5, per 15 min	1.08 (1.03–1.13)	<0.001	1.17 (1.09–1.26)	<0.001	1.1 (1.02–1.18)	0.01
PPI ≤1.5, per 15 min	1.04 (1.01–1.07)	0.01	1.08 (1.04–1.12)	<0.001	1.05 (0.99–1.11)	0.1
Mean MAP ≤65, yes	1.21 (0.92–1.58)	0.2	0.79 (0.57–1.09)	0.2	0.8 (0.47–1.33)	0.4
MAP ≤65, per 15 min	1.04 (1–1.08)	0.04	1.03 (1–1.08)	0.2	1.02 (0.95–1.1)	0.6
Surgery, AHA	3.74 (2.89–4.87)	<0.001	3.36 (2.39–4.79)	<0.001	–	–
Infusion vasopressor, yes	2.49 (1.93–3.21)	<0.001	2.29 (1.67–3.14)	<0.001	1.36 (0.77–2.33)	0.3
Fluids∗, per 100 ml	1.07 (1.05–1.08)	<0.001	1.06 (1.05–1.08)	<0.001	1.01 (1–1.05)	0.7
Blood loss, per 100 ml	1.06 (1.04–1.09)	<0.001	1.06 (1.03–1.1)	<0.001	1.07 (1.02–1.14)	1.01
Age, per yr	1 (1–1.01)	0.9	1 (0.99–1.01)	0.7	1.03 (1.01–1.06)	0.001
Sex, male	1.31 (1.02–1.69)	0.03	1.19 (0.87–1.62)	0.3	1.12 (0.88–2.27)	0.2
ASA physical status ≥3, yes	2.05 (1.58–2.68)	<0.001	1.74 (1.27–2.4)	0.001	2.96 (1.78–5.11)	<0.001
Zubrod score ≥III, yes	2.6 (1.83–3.68)	<0.001	2.14 (1.41–3.23)	<0.001	3.16 (1.59–6.05)	0.001
CCI, per 1 increase in index	1.14 (1.08–1.2)	<0.001	1.1 (1.04–1.16)	0.001	1.28 (1.15–1.43)	<0.001

Odds ratio (95% confidence interval). AHA, acute high-risk abdominal surgery; ASA, American Society of Anesthesiologists; CCI, Charlson Comorbidity Index; PPI, peripheral perfusion index. ∗Ringer's acetate, Ringer's lactate, NaCl, HA, Voluven®, or any combination of these.

In the multivariable model assessing the association of PPI with the primary outcome (Clavien–Dindo Class ≥III) and adjusting for confounding factors as age, sex, type of surgery, type of anaesthesia, infusion of (any) vasoactive medication, administered fluids, blood loss, and comorbidities, OR was 1.12 (95% CI 0.05–1.19; *P*<0.001). The multivariable association of PPI ≤0.5 and PPI ≤1.5 with the primary outcome was 1.79 (95% CI 1.09–2.91; *P*=0.02) and 1.65 (95% CI 1.20–2.27; *P*=0.002), respectively. Univariable and multivariable estimates for the association of PPI with Clavien–Dindo Class ≥III and 30-day mortality are displayed in Table 3. Estimates for the multivariable model are displayed in Supplementary Table S1.

**Table 3:** Univariable and multivariable association of PPI with severe postoperative complications Clavien–Dindo Class ≥III within 30 days and 30-day mortality in acute high-risk abdominal and hip fracture patients.

**Postoperative complications**	**Unadjusted OR (95% CI)**	***P***	**Adjusted∗ OR (95% CI)**	***P***
PPI, per 1.0 decrease in mean PPI	1.07 (1.02–1.13)	0.01	1.12 (1.05–1.19)	<0.001
Mean PPI ≤0.5, yes	1.67 (1.08–2.55)	0.02	1.79 (1.09–2.91)	0.02
Mean PPI ≤1.5, yes	1.36 (1.04–1.78)	0.002	1.65 (1.2–2.27)	0.002
PPI ≤0.5, per 15 min	1.08 (1.03–1.13)	<0.001	1.11 (1.05–1.17)	<0.001
PPI ≤1.5, per 15 min	1.04 (1.01–1.07)	0.01	1.06 (1.02–1.09)	0.002
**Thirty-day mortality**	**Unadjusted OR (95% CI)**	***P***	**Adjusted∗ OR (95% CI)**	***P***
PPI, per 1.0 decrease in mean PPI	1.25 (1.14–1.37)	<0.001	1.2 (1.08–1.34)	0.001
Mean PPI ≤0.5, yes	2.25 (1.3–3.74	0.002	1.94 (1.04–3.48)	0.03
Mean PPI ≤1.5, yes	2.21 (1.55–3.16)	<0.001	2.07 (1.37–3.14)	0.001
PPI ≤0.5, per 15 min	1.16 (1.1–1.22)	<0.001	1.16 (1.09–1.24)	<0.001
PPI ≤1.5, per 15 min	1.09 (1.05–1.14)	<0.001	1.09 (1.04–1.14)	<0.001

∗Adjusted for: comorbidities; ASA physical status ≥3, Zubrod score ≥III, Charlson Comorbidity Index, age, sex, type of anaesthesia, type of surgery, i.v. fluid administration, blood loss, MAP ≤65 mm Hg. CI, confidence interval; OR, odds ratio.

### Effect of intraoperative time spend under PPI thresholds

**Fig 3:**
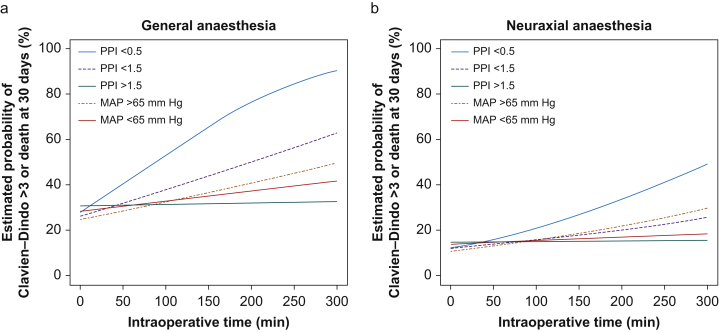
Effect of time below/above predetermined thresholds of mean intraoperative peripheral perfusion index in patients undergoing (a) general anaesthesia and (b) neuraxial anaesthesia.

In univariable models evaluating the association of time (15 min increments) spend under prespecified thresholds of very low (≤0.5) and low (≤1.5) values of PPI, OR were 1.08 (95% CI 1.03–1.13; *P*<0.001) and 1.04 (95% CI 1.01–1.07; *P*=0.01), respectively (Table 3). The estimated probabilities of severe postoperative complications or death according to cumulated time below and above PPI and MAP thresholds are presented in Fig 3, with PPI >1.5 presenting with the lowest probability of negative outcome and PPI ≤0.5 with the highest probability regardless of neuraxial *vs* general anaesthesia.

### Interaction with MAP

MAP ≤65 mm Hg was not associated with the primary outcome in the univariable analysis and we found no significant confounding of MAP ≤65 mm Hg on the association of PPI with the primary outcome in the multivariable model (Table 3). However, including the interaction between PPI and MAP ≤65 mm Hg, OR for the association of PPI with the primary outcome was increased from 1.07 (95% CI 1.02–1.13; *P*=0.011) (crude) to 1.17 (95% CI 1.09–1.27; *P*<0.001) in patients with MAP ≤65 mm Hg compared with patients with MAP >65 mm Hg.

### PPI cut-off evaluation

ROC curve analysis of the association of PPI with the primary outcome yielded an AUC of 0.56 (0.52–0.59) and maximising sensitivity and specificity using Youden's index yielded a best cut-off value of mean intraoperative PPI 2.7 with a sensitivity of 0.51 and a specificity of 0.6. A cut-off of mean intraoperative PPI 1.5 yielded a sensitivity of 0.72 and a specificity of 0.37.

## Discussion

In this retrospective, multicentre, cohort study of acute surgical patients with detailed intraoperative haemodynamic information, we found that intraoperative PPI was significantly associated with severe postoperative complications and mortality, with OR increasing by 12% with each absolute decrease by 1.0 in intraoperative PPI. Intraoperative hypotension defined as MAP ≤65 mm Hg was not independently associated with severe outcomes and we did not find any modifying effect of hypotension on the effect of PPI on outcome. However, there was a significant interaction between PPI and MAP with a 10% increase in the association of PPI with outcome in patients with hypotension, suggesting a synergistic effect of hypotension in patients with poor perfusion, but also that hypotension may be of little consequence in patients with adequate perfusion. Additionally, the probability of severe postoperative complications or death within 30 days was associated with the cumulative intraoperative time patients spend with a low PPI, and PPI >1.5 appears to present the lowest and PPI <0.5 the highest probability of a negative outcome. The 30-day mortality in patients presenting PPI ≤0.5 was twice as frequent as in patients presenting PPI >0.5. This could suggest that avoiding poor peripheral perfusion may lead to improved survival.

Perioperative haemodynamic optimisation is based on cardiovascular monitoring, with the target being tissue perfusion.^25^ The GDT concept has emerged as an individualised approach to optimise flow parameters, which has gained prominence by the advent of minimally invasive cardiovascular monitoring. Yet in most settings, MAP is the primary resuscitation target.^26^ Recent large studies report strong associations between even short periods of intraoperative hypotension and postoperative morbidity and mortality.^27^ However, intervention studies with focus on avoiding hypotension as a resuscitation goal *per se* have not resulted in improved outcome.^28^ In contrast, studies on strict BP control after optimisation of perfusion within a GDT protocol have shown promise^29^ and results from large trials on optimisation of flow are pending.^4^^,^^5^ We consider findings of the present study important since postoperative morbidity and mortality continue to be high in acute surgical patients.^17^ At present, focus on prevention of hypotension receives attention^30^ and yet the means of preventing/correcting hypotension and the consequent association with postoperative outcome are inadequately understood.^31^ Our data suggest that low PPI is an important perioperative indicator that alone, or in tandem with hypotension, may contribute to poor postoperative outcome. Results are consistent with studies of perioperative PPI, where lower PPI is associated with serious adverse events.^15^^,^^32^

Sympathetic, and thus vascular tone is a major determinant of PPI. Induction and maintenance of anaesthesia suppress sympathetic tone in a dose-dependent manner facilitating vasoplegia, possibly inducing or increasing preload dependency but also affecting PPI, which reflects the ratio between the pulsatile and non-pulsatile component of the arterial waveform in the observed tissue. However, associations of low PPI with outcome are consistent regardless of type of anaesthesia, probably reflecting that both sympathetic activity and low CO are major determinants of PPI.

Maintaining MAP >65 mm Hg presented a higher probability of poor outcome than presumed hypotensive states (MAP ≤65 mm Hg) and presumed adequate perfusion (PPI >1.5; Fig. 3). This finding is contrary to data from other studies that found temporal associations between hypotension and outcome.^27^ This discrepancy may be attributable to the type of high-risk patients included in the cohort, who because of the nature of their pathology, may have a high degree of dissociation between macrocirculation and microcirculation. Enhancing afterload with vasopressors targeting MAP >65 mm Hg without assessing whether hypotension is attributable to high-flow or low-flow states might compromise perfusion, and thus lead to poorer outcomes. Close monitoring of PPI, which is obtained noninvasively and continuously by photoplethysmography, universal in the perioperative setting, may be a feasible haemodynamic monitoring modality. Potentially, the addition of perfusion parameters to GDT algorithms may be instrumental in guiding the clinician to the correct intervention.^31^

Although we adjusted our analysis for potential confounding factors, it is possible that some of the observed association between PPI and poor postoperative outcome could be attributable to unobserved confounding. However, it seems most likely, because of consistent significant association and dose–response profile, that this association represents a causal relationship. If so, that is interesting because PPI is highly modifiable by intraoperative haemodynamic interventions and therefore not a static predictor, and as such interventions that increase PPI could improve outcome.^33^^,^^34^ Yet, causal relationship between interventions targeting PPI and outcome can only be established in randomised trials. The strength of the overall association with morbidity and mortality would seem to justify a major trial, although only after the potential of different interventions to optimise circulation and perfusion, as measured by PPI, have been further investigated. In the interim, and given the modest predictive utility of PPI, clinicians might be prudent to assume that PPI <0.5 could be detrimental to patient outcome and should prompt consideration of treatment aimed at resuscitating tissue perfusion.

A strength of this analysis is the sample size derived from two centres with a high flow of acute surgical patients. We included detailed and consistent data on PPI and MAP, with absolute values sampled every 15 min throughout the intraoperative period, and missing data did not exceed 10%. Data were collected retrospectively, but hypothesis, endpoints, and statistical analysis plan were peer-reviewed and published before the data collection was initiated.^21^

In conclusion, low values of intraoperative PPI, and the duretion of intraoperative time spend with low values of PPI, were associated with severe postoperative complications or death in acute high-rsik surgical patinets. The duration of intraoperative low values of PPI were also. Further interventional studies targeting PPI thresholds may help in identifying strategies that may reduce severe postoperative complications in acute noncardiac surgery.

## Authors' contributions

Participated in the study concept and design: MA, HS, CSM, SW, NBF

Conducted the construction of the database and data entry: MA, ANWT

Participated in the study conduct, data analysis, and writing of the manuscript: MA, HS, JH, CSM, NHS, SW, NBF

Read and approved the final manuscript: all authors

All the above fulfil the Vancouver principles to be granted authorship.
