# Erratum to ‘Association of the intraoperative peripheral perfusion index with postoperative morbidity and mortality in acute surgical patients: a retrospective observational multicentre cohort study’ (*Br J Anaesth* 2021; 127: 396-404)

**DOI:** 10.1016/j.bja.2022.01.003

**Published:** 2022-01-20

**Authors:** Marianne Agerskov, Anna N.W. Thusholdt, Henrik Holm-Sørensen, Sebastian Wiberg, Christian S. Meyhoff, Jakob Højlund, Niels H. Secher, Nicolai B. Foss

**Affiliations:** 1 Department of Anaesthesiology and Intensive Care, Hvidovre Hospital, University of Copenhagen, Copenhagen, Denmark; 2 Department of Integrative Physiology, NEXS, University of Copenhagen, Copenhagen, Denmark; 3 Department of Anaesthesia and Intensive Care, Bispebjerg and Frederiksberg Hospital, University of Copenhagen, Copenhagen, Denmark; 4 Department of Anaesthesiology, Centre for Cancer and Organ Diseases, Rigshospitalet, University of Copenhagen, Copenhagen, Denmark

The publisher regrets that an error was present in Figure 2. The corrected figure is shown below:

**Figure:**
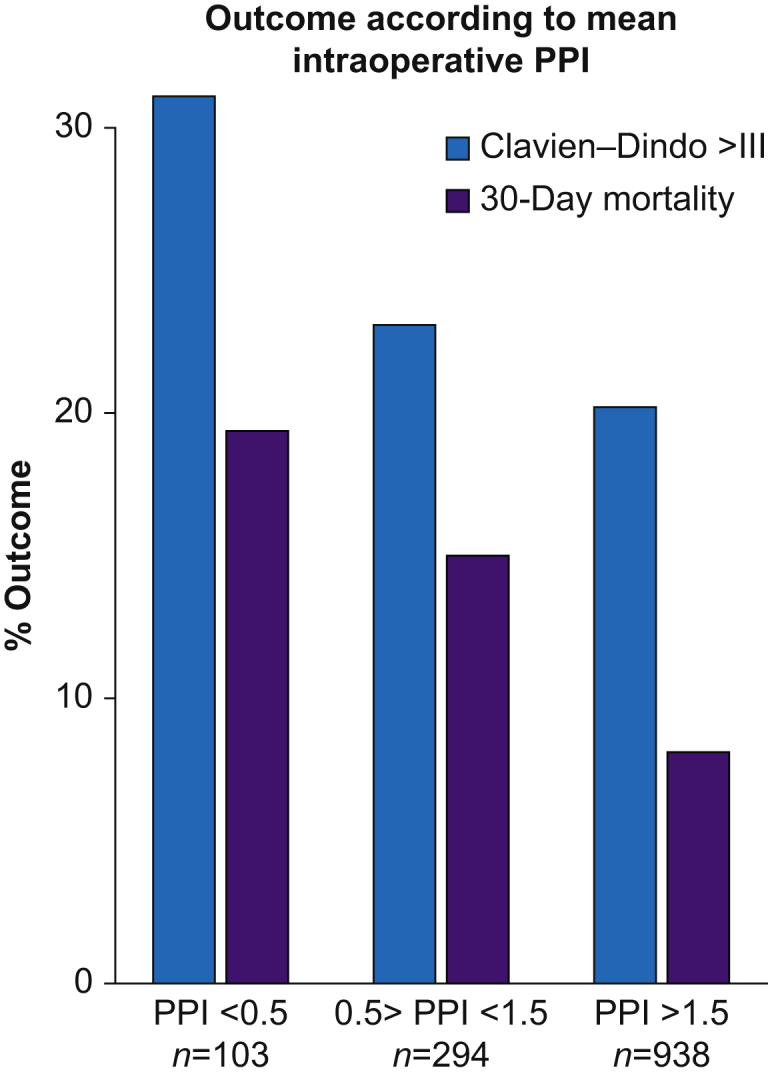

The publisher would like to apologise for any inconvenience caused.

