# Supplementary material for: Association of the intraoperative peripheral perfusion index with postoperative morbidity and mortality in acute surgical patients: a retrospective observational multicentre cohort study
Source: Br J Anaesth. 2021 Jul 3;127(3):396–404. doi: 10.1016/j.bja.2021.06.004 (PMC8451236; doi:10.1016/j.bja.2021.06.004)
Supplement: Multimedia component 1 [file mmc1.docx]

**Appendix Table 1:** Full multivariable logistic regression model of the association of the primary exposure variable PPI and risk factors/confounders with the primary outcome of severe postoperative complications

Clavien Dindo Class≥ III within 30 days in acute high-risk abdominal and hip fracture patients

|  | **OR (95% CI)** | **p** |
| --- | --- | --- |
| **PPI, per 1.0 decrease in mean PPI** | 1.12 (1.05 – 1.19) | <0.001 |
| **Mean MAP≤ 65, yes** | 0.96 (0.71 – 1.31) | 0.8 |
| **Surgery, AHA** | 4.79 (3.1 – 7.51) | <0.001 |
| **Type of anaesthesia, GA** | 1.1 (0.73 – 1.67) | 0.6 |
| **Infusion vasopressor, yes** | 1.26 (0.92 – 1.73) | 0.1 |
| **Fluids˜, per 100 mL** | 1.02 (1 – 1.04) | 0.01 |
| **Blood loss, per 100 mL** | 1.06 (1.02 – 1.09) | 0.001 |
| **Age, per year** | 1 (1 – 1.02) | 0.5 |
| **Gender, male** | 0.78 (0.58 – 1.04) | 0.1 |
| **ASA class≥ III, yes** | 1.61 (1.14 – 2.29) | 0.01 |
| **Zubrod score≥ III, yes** | 1.94 (1.29 – 2.89) | 0.001 |
| **CCI, per 1 increase in index** | 1.08 (1.01 – 1.16) | 0.03 |

OR: Odds Ratio, CI: Confidence Interval, PPI: Peripheral Perfusion Index, MAP: Mean Arterial Pressure, AHA: Acute High-risk Abdominal surgery,

GA: General anaesthesia, ASA: American Association of Anesthesiologists, CCI: Charlson Comorbidity Index.

˜Ringers Acetate and/or Ringers Lactate and/or NaCl and/or HA and/or Voluven®.
